# To Evaluate the Effect of Different Adhesive Materials on the Microleakage of Bonded Amalgam Restorations: An *in vitro* Study

**DOI:** 10.5005/jp-journals-10005-1197

**Published:** 2013-08-26

**Authors:** Sumit Bembi, Nitika Bembi Narula, Amit Sood, Amarjeet Gambhir

**Affiliations:** Reader, Department of Conservative Dentistry and Endodontics, SKSS Dental College, Ludhiana, Punjab, India, e-mail: s_bembi@yahoo.com; Senior Lecturer, Department of Periodontology, SKSS Dental College Ludhiana, Punjab, India; Reader, Department of Conservative Dentistry and Endodontics, SKSS Dental College, Ludhiana, Punjab, India; Reader, Department of Oral and Maxillofacial Surgery, SKSS Dental College, Ludhiana, Punjab, India

**Keywords:** Amalgam, Microleakage, Dentin adhesives, Copal varnish

## Abstract

This study evaluated the ability of different adhesive materials in reducing the microleakage in class V amalgam restorations. Standardized class V cavities were prepared on the facial surface of 56 noncarious human premolars, they were then randomly divided into control and experimental groups based on adhesives used. Group I was the control group with copal varnish, group II had Panavia F 2.0, group III contained Vitrebond Plus and group IV had RelyX ARC as adhesives. Amalgam was hand condensed into each preparation after application of adhesive material. Specimens were thermocycled, stained and sectioned. Microleakage was graded using a stereomicroscope. Less leakage was observed in all experimental groups compared to control group (p < 0.01) on nonparametric Kruskal-Wallis test. Mann-Whitney test observed leakage was more extensive at the gingival margins (p < 0.01) in all restorations than at occlusal margins. Group III showed no leakage which was significantly different from other groups (p < 0.05). Hence, this study concluded that application of intermediate adhesive material before condensation of amalgam can act as an effective barrier for microleakage.

**How to cite this article:** Bembi S, Bembi NN, Sood A, Gambhir A. To Evaluate the Effect of Different Adhesive Materials on the Microleakage of Bonded Amalgam Restorations: An *in vitro* Study. Int J Clin Pediatr Dent 2013;6(2):95-99.

## INTRODUCTION

Dental amalgam has been an age old direct restorative material used in dentistry. It is one of the least technique sensitive materials which is highly resistant, insoluble in oral fluids, inexpensive and tolerates a great deal of misuse without obvious failures. However, apart from its controversy about mercury toxicity, amalgam has had its own disadvantages in particular of microleakage and lack of adhesion which makes undercuts necessary for its mechanical retention thereby further weakening remaining tooth structure. Microleakage can cause hypersensitivity of restored tooth, tooth discoloration, recurrent caries, even pulpal injury and accelerated deterioration of material. Conventional amalgam alloys displays a marked decrease in microleakage as restoration ages due to sealing by corrosion products formed by gamma II tin-mercury phase which is the weakest phase of set amalgam. However, newer high copper amalgam restorations for optimized clinical handling and performance are usually free of the most corrosive gamma II phase. As a result less corrosive products are created with high copper amalgam because of slower corrosion process than for conventional amalgams. It was then thought that the use of another material between the tooth and amalgam may help to overcome this problem by creating a seal and may also improve the retention of the material.^1^ Many materials have been employed to fill the amalgam tooth interface and improve retention by bonding. These have included zinc phosphate cement, copalex varnish and polycarboxylate cement. Since, the mid-1980s, resin composite adhesives which bond to metal have been used with the aim of forming a bond between amalgam and tooth structure known as bonded amalgam. This bond is not merely mechanical but it includes a molecular interaction. These bonded amalgam restorations seem to provide substantial amalgam retention ability without compromising on tooth structure, reducing microleakage, cusp flexure and initial postoperative sensitivity. Resin-based composites, either setting by a dual cure or chemical (anaerobic) reaction, have also been used for this purpose, as have resin-modified glass polyalkenoate (ionomer) cements.^2,3^ However, these different adhesive materials employed in bonded amalgam technique have produced different sealing abilities.^4,5^ Thus, the purpose of this study was to evaluate the effect of new adhesive materials on the microleakage of bonded amalgam restorations.

## MATERIALS AND METHODS

A total of 56 recently extracted noncarious human premolars were selected and stored in 0.1% thymol solution at room temperature for up to 4 weeks. Teeth were then consecutively debrided with slurry of pumice flour and examined to ensure absence of any defects. Standardized class V cavities were prepared on the facial surface of each tooth (3 mm length, 2 mm deep, 2 mm wide) having gingival margins in cementum/dentin and occlusal margins in enamel, using a #245 bur with a high speed handpiece and copious amount of water. A new bur was used after every five cavity preparations to ensure high cutting efficiency. Each preparation was cleaned with air water spray from triple syringe for 10 seconds and air dried. The teeth were then randomly divided into four experimental groups (n = 16) according to adhesive used under amalgam restoration as follows:

Group I copalex varnish (Kuraray Medical Inc, Okayama, Japan): The varnish was applied in two thin layers, allowing the first layer to air dry for 30 seconds prior to applying the second layer.

Group II Panavia F 2.0 (Kuraray Medical Inc, Okayama, Japan): Enamel was etched with phosphoric acid. After washing and drying one drop each of ED primer liquid A and B were mixed and applied over the enamel and dentin for 60 seconds. Surface was gently air dried for 1 second. A fine coat of Panavia F 2.0 was applied to entire cavity using a brush, having mixed, previously, two pastes of system for 20 to 30 seconds.

Group III Vitrebond Plus (3M ESPE, St. Paul, MN USA): Cavities were conditioned with vitremer primer, which remained in place for 30 seconds followed by photocuring for 20 seconds. Vitrebond Plus was mixed and a thin layer of material was applied on cavity walls.

Group IV RelyX ARC (3M ESPE, St. Paul, MN USA): Enamel and dentin were acid etched with 37% phosphoric acid for 15 seconds. Cavities were rinsed for 10 seconds with water and blot dried with a cotton pellet to remove excess water and to avoid desiccation. Two consecutive coats of 3M ESPE Adper Single Bond adhesive was applied and light cured for 10 seconds. RelyX ARC cement was mixed and a thin layer was applied in cavities using a brush.

Following application of these adhesives, amalgam [admixed dispersed phase alloy powder (Dentsply India Pvt Ltd.) triturated with mercury (Deepak Enterprises, Mumbai, India) in amalgamator (Dentomat, Degussa, Brazil)] was condensed in horizontal increments using ward and hollenback condensers into the cavities before setting of the adhesive materials. Amalgam was condensed in horizontal increments using ward and hollenback condensers before setting of the adhesive materials. In group II after placing amalgam restorations oxyguard (included in panavia kit) was applied on all marginal areas with a brush and removed after 3 minutes. Carving was performed using 3S hollen back carver. All the restored teeth were stored in distilled water for 7 days before finishing and polishing.

## MICROLEAKAGE ASSESSMENT

**Fig. 1:**
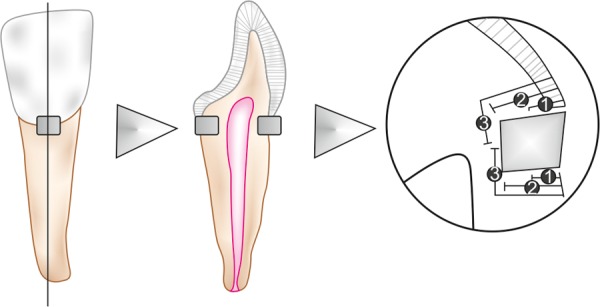
Restoration diagrams and evaluation scores: 0–no dye penetration; 1–dye penetration up to 1/3 of cavity depth; 2–dye penetration up to 2/3 of cavity depth; 3–dye penetration up to the cavity floor

After storage, the restored teeth were thermocycled for 500 cycles between 5 and 55^°^C with a dwell time of 1 minute. The samples were then blotted dry with a paper towel and the root apexes of each tooth sealed with resin composite (3M ESPE, St Paul, MN, USA). An acid resistant varnish (finger nail polish) was applied to all the surfaces of the teeth except for the restorations and 1 mm surrounding them. Specimens were then immersed in 1% methylene blue dye for 24 hours at room temperature. After 24 hours they were removed, thoroughly rinsed and the nail polish was gently removed with a sterile #15 disposable scalpel blade (Lister, India). The teeth were then embedded in acrylic autopolymerizing resin and labeled. A low speed diamond disk under constant water irrigation was used to section each tooth block longitudinally through the centre of restoration from buccal to lingual surface. Dye penetration was assessed under magnification 40× using a calibrated stereo-microscope and scored as shown in Figure 1 in which:

*Score 0:* No dye penetration

*Score 1:* Dye penetration up to one-third of the cavity depth

*Score 2:* Dye penetration up to two-third of the cavity depth

*Score 3:* Dye penetration up to cavity floor.

Two readings (averaged) were taken at enamel and cementum/dentin margins of each tooth blocks. Leakage data obtained was statistically analyzed using nonparametric Kruskal-Wallis test with confidence levels set at 95% and Mann-Whitney tests.

## RESULTS

**Fig. 2:**
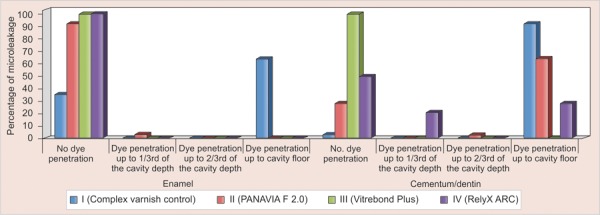
Percentage frequency of microleakage occurring at the enamel margin and cementum/dentin margins of amalgam restorations

Dye penetration (leakage) scores of all experimental groups in enamel and cementum/dentin are presented in Table. 1. Significant differences in microleakage between the enamel and dentin margins were observed, being statistically greater (p < 0.01) at the cementum/dentin margin than found in enamel for all groups (Mann-Whitney test) (Fig. 2). Vitrebond Plus group showed significantly less leakage (p < 0.05) than control and other adhesive groups when results from enamel and cementum/dentin were taken together (Kruskal-Wallis test). Similar results were observed for remaining adhesive material demonstrating significantly less leakage (p < 0.01) than the control group.

All adhesive materials showed similar leakage in enamel, being statistically less than control group (p < 0.01). Within dentin margin (Fig. 2) Vitrebond Plus showed less leakage than all other groups (p < 0.01). Both Panavia F 2.0 and RelyX ARC showed less leakage than control group (p < 0.01). Panavia F 2.0 exhibited greater leakage than RelyX ARC (p < 0.05). No specimen from the Vitrebond Plus group exhibited dye penetration in either enamel or dentin/cementum margins (Fig. 2).

## DISCUSSION

Prevention of microleakage is an important aspect for longevity of restorations. Microleakage is defined as the clinically undetectable passage of bacteria, fluids, molecules or ions from the oral environment along the various gaps present in between a cavity wall and the material applied to it. Its clinical relevance is that the passage of bacteria at the tooth restoration interface may cause recurrent caries or pulpal irritation with subsequent pulpal inflammation.^6^ This could lead to a clinical diagnosis of reversible pulpitis or loss of vitality of the tooth. Operative intervention would then be necessary which, at best, would require a replacement restoration with the cavity inevitably increasing in size or, at worst, endodontics or extraction of the tooth. Many techniques have been used for assessment of microleakage in dental restorations. In this study, dye penetration test was chosen because it provided a simple, relatively cheap, qualitative and comparable method of evaluating the performance of the various restoration techniques.^7^ Thermocycling was performed as it was thought that this simulates the condition of restoration in oral cavity in terms of predicting the *in vivo* performance of restorations, since thermal stresses and water exposure continuously work on restorations. Thus, it provides important information on the possible clinical performance of new materials. Methylene blue dye was used to evaluate microleakage because it is simple and inexpensive with better penetration results than eosin or other radioisotope traces.

Various adhesive liners and cavity varnishes have been used as methods to decrease microleakage around a fresh amalgam restoration.^8^ However, varnishes were found to only serve as a mechanical barrier, did not bond to the amalgam or the tooth structure and dissolved by the passage of time with its long-term seal still a concern.^9^ Studies comparing resin-lined and varnish-lined amalgams concluded that bonded amalgam leaked less than varnish-lined restorations. The results of present study were in accordance to these studies wherein adhesive materials used as liner showed less leakage then copal varnish.^10,14^ Few studies though also concluded that these agents should not be used routinely to control microleakage, as increased dye penetration was observed.^11^

**Table 1:** **Table 1:** Dye penetration scores in enamel and cementum/dentin interfaces of experimental groups (n = 28/group)

Groups		*Enamel*										*Cementum/Dentin*					
		0		1		2		3		0		1		2		3	
I (Copalex varnish control)		10		0		0		18		2		0		0		26	
II (PANAVIA F 2.0)		26		2		0		0		8		0		2		18	
III (Vitrebond Plus)		28		0		0		0		28		0		0		0	
IV (RelyX ARC)		28		0		0		0		14		6		0		8	

This study observed overall significantly less microleakage at the occlusal margins of cavity than that at the gingival margin in **c**ongruence to the findings reported by other investigators.^1,12^ This could be due to wider surface area available for bonding at the occlusal margin having enamel than at gingival where the margin is comprised of dentin. The unique characteristics of the dentin substrates, including high organic content, low calcium concentration, tubular structure variations, and the presence of outward fluid movement may have adversely affected the adhesion in dentin.^13^ Furthermore, permeability of dentin relative to enamel and the small size of methylene blue particles could have also led to higher penetration of the dye at gingival margins.

In this study Vitrebond Plus a resin-modified glass ionomer (RMGIC) used as an intermediate material was effective in eliminating dye penetration in all specimens which was in accordance with other studies.^14^ RMGICs are glass-ionomer cements with the incorporation of a small quantity of monomers as well as initiators involved in the polymerization reaction. RMGIC has dual setting reaction consisting of fundamental acid-base curing reaction supplemented by a second polymerizable reaction either induced chemically or by visible light. Improved adhesion to dentin is probably caused by both a chemical bonding from the polyacrylic acid component and micromechanical interlocking achieved by formation of a hybrid layer from the hydrophilic HEMA. In the present study, Vitrebond was applied following treatment of the cavity with the vitremer primer, which is not a recommendation of the manufacturer. Such application was based on several studies in which previous treatment of tooth substrate increased adhesion of glass ionomer.^15^ It is possible that the pH of the dentin primer could modify the smear layer sufficiently to permit the tooth and restorative material to come into intimate interfacial contact contributing to the improved performance of this technique in the present study.

Microleakage in enamel was similar for both resin cements which could be due to enamel etching with phosphoric acid and the higher mineral content of the tissue might allow better sealing. Panavia F 2.0 combined with the self-etching adhesive, ED Primer, showed significantly higher microleakage values than Vitrebond and RelyX ARC in dentin margins. This can be explained by different bonding mechanisms of the total-etch and the self-etching techniques. Panavia F 2.0 (Kuraray Medical Inc, Tokyo, Japan) is an example of a one-step self-etch, self-adhesive, dual cure fluoride releasing resin cement in which the resin cement is coupled to primed enamel and dentine. RelyX ARC is a total etch adhesive involving a separate etch and rinse step followed application of hydrophilic dentin bonding agent. One-step self-etch adhesives, because of their higher concentrations of hydrophilic and ionic resin monomers behave as permeable membranes after polymerization.^16^ The increase in permeability in one-step self-etch adhesives allows water to diffuse from dentin across the polymerized adhesive and form water droplets along the adhesive–composite interface. Moreover, the inclusion of acidic monomers in self-etch reduces the rate and extent of polymerization, this slow polymerization rate of Panavia F 2.0 may allow more water to diffuse from the vital dentin into the hydrophilic interface between the Panavia F 2.0 primer and dentin, due to its more hydrophibic nature. Apparently, early water exposure of self-etching, slow-curing primers or adhesives, as was done in this study by storage of specimens in distilled water, also does compromise their mechanical properties due to plasticization of the polymer molecules could have also added to more leakage.

Major limitations of this study included usage of only thermoclycing to age and stress the restored tooth, with no load cycling applied, not taking into consideration the effect of repeated load cycling within the physiologic chewing range on resin-bonded restorations. Moreover, the storage time used in this study was not long and longevity of the adhesive bond strength remains an important question. *In vitro* tests have been used to study some properties of materials to provide information about their potential clinical performance. However, *in vitro* tests cannot adequately simulate clinical conditions. So results of *in vitro* tests should be applied with caution to the clinical situation only after being substantiated by *in vivo* evidence requiring long-term clinical studies. Thus further studies are required in order to evaluate the role of these materials in reducing leakage when used with amalgam restorations.

## CONCLUSION

Within the limitation of this study, data analysis observed that microleakage was less at both enamel and dentin margins of amalgam restorations treated with adhesive resin systems compared to those amalgam restorations lined with the conventional Copalite Varnish. However, leakage at enamel margins was significantly less than at dentin margins with usage of adhesive liner. In dentin margins Panavia F 2.0 showed more leakage than RelyX ARC though both had less leakage than the control group. Vitrebond Plus provided total prevention of microleakage in all specimens. Thus, it can be concluded that usage of adhesive liners with amalgam restorations showed better results in dye penetration prevention. But much more work is required, ideally with the execution of prospective, randomized, controlled clinical studies, over a long period of time, to determine the longevity and success rate of such bonded amalgam restorations.
